# Effects of Water Deficits on *Prosopis tamarugo* Growth, Water Status and Stomata Functioning

**DOI:** 10.3390/plants10010053

**Published:** 2020-12-29

**Authors:** Alson Time, Edmundo Acevedo

**Affiliations:** 1 Programa Magister en Ciencias Agropecuarias, Facultad de Ciencias Agronómicas, Universidad de Chile, Santa Rosa, La Pintana, Santiago 11315, Chile; 2 Laboratory Relation Soil-Water-Plant (SAP), Department of Agricultural Production, Faculty of Agronomic Sciences, University of Chile, Santiago 1004, Chile

**Keywords:** water potential, drought, water depletion, twig elongation

## Abstract

The effect of water deficit on growth, water status and stomatal functioning of *Prosopis tamarugo* was investigated under controlled water conditions. The study was done at the Antumapu Experiment Station of the University of Chile. Three levels of water stress were tested: (i) well-watered (WW), (ii) medium stress intensity (low-watered (LW)) and (iii) intense stress (non-watered (NW)), with 10 replicates each level. All growth parameters evaluated, such as twig growth, specific leaf area and apical dominance index, were significantly decreased under water deficit. Tamarugo twig growth decreased along with twig water potential. The stomatal conductance and CO_2_ assimilation decreased significantly under the water deficit condition. Tamarugo maintained a high stomatal conductance at low leaf water potential. In addition, tamarugo reduced its leaf area as a strategy to diminish the water demand. These results suggest that, despite a significant decrease in water status, tamarugo can maintain its growth at low leaf water potential and can tolerate intense water deficit due to a partial stomatal closing strategy that allows the sustaining of CO_2_ assimilation in the condition of reduced water availability.

## 1. Introduction

Water is one of the most limiting factors for plant growth. Thus, for fighting stress caused by water limitation, plants have developed mechanisms that allow them to cope with drought caused by water shortage. In presence of environmental stress whose influences exceeds the resistance limit for the survival of the woody plants, the plants could activate some physiological and biochemical mechanisms that allow them to resist those stressors. Nevertheless, that resistance may depend on the flexibility of these mechanisms, the compensatory abilities, the intensity and the duration of the stressors [1]. Presently, a great number of forest plants’ biomes have experienced an increasing mortality rate, which is associated with rises in temperature and a greater incidence of drought [2]. Human activities can also produce stresses in the future that can significantly affect the already stressed environment [3]. This is the situation of the Pampa del Tamarugal (Atacama Desert, Chile), which is described as a hyper-arid desert mostly populated by *Prosopis tamarugo*. Tamarugo, which is a strict phreatophyte plant, is growing under a water table reduction condition due to groundwater extraction for the mining industry, agriculture supply and urban areas [4].

The effects of water deficit on plants are well studied. Reports demonstrate that water deficit can affect several variables and functions in plants, such as stomata functioning [5], hydric traits such as water potentials and xylem hydraulic conductivity [6,7,8] and growth traits like twig growth, specific leaf area and leaf shoot ratio [9,10]. The functioning of these parameters and other processes may determine the growth capacity and the survival of the plant [11].

The regulation of stomata in plants determines the consumption of and assimilation of CO_2_ and can influence the ability of plants to survive extreme conditions of water stress. In a water stress condition due to drought, the opening of the stomata is affected by the soil and plant water content. It has been demonstrated that stomata remain unaffected until the leaf water potential drops to some critical threshold value [12]. The closure of stomata in a water stress situation is considered one of the main factors that limit photosynthesis [13]. However, this depends on the plant’s stomatal behavior [11], as plants fall into two categories: isohydric and anisohydric. Under water stress, isohydric plants tend to close their stomata earlier than anisohydric plants, which delay their stomata closure, as well as enhance gas exchange and let the leaf water potential decrease as the soil water potential decreases [5,14]. Isohydric plants generally experience a negative carbon balance, while anisohydric plants experiment low water potential, which may provoke hydraulic failure by xylem cavitation and embolism.

*Prosopis tamarugo*, a desert plant, has developed several mechanisms of adaptation that allow survival in the extreme environmental conditions where it grows. There are reports of mechanisms such as the capacity to maintain high stomata conductance when subjected to increasing temperature and atmospheric water demand [15,16], partial closure of stomata to decrease water loss, angle changes of its leaflets to avoid high levels of radiation in the afternoon [17] and osmoregulation, which is a solute accumulation in the cells allowing a positive turgor pressure development in spite of low water potential.

Studying the aboveground growth response of tamarugo to the intensity and duration of water stress is of interest, as it may lead to guaranteeing the survival of tamarugo and save it from extermination provoked by induced groundwater depletion.

Growth of tamarugo was studied under the semi-controlled condition in the Antumapu Experiment Station of the Faculty of Agronomy of the University of Chile during the spring of 2016, with the objective of determining the effects of the water stress on the water status growth and the stomata functioning of *P. tamarugo*.

## 2. Results

### 2.1. Effect of Water Stress on Tamarugo Growth, Isotopic Discrimination of ^13^C and Enrichment of ^18^O

A clear significant difference was observed for the twig growth rate in all watering levels. The well-watered plants had a high growth rate in all sampling dates (Table 1).

**Figure 1:**
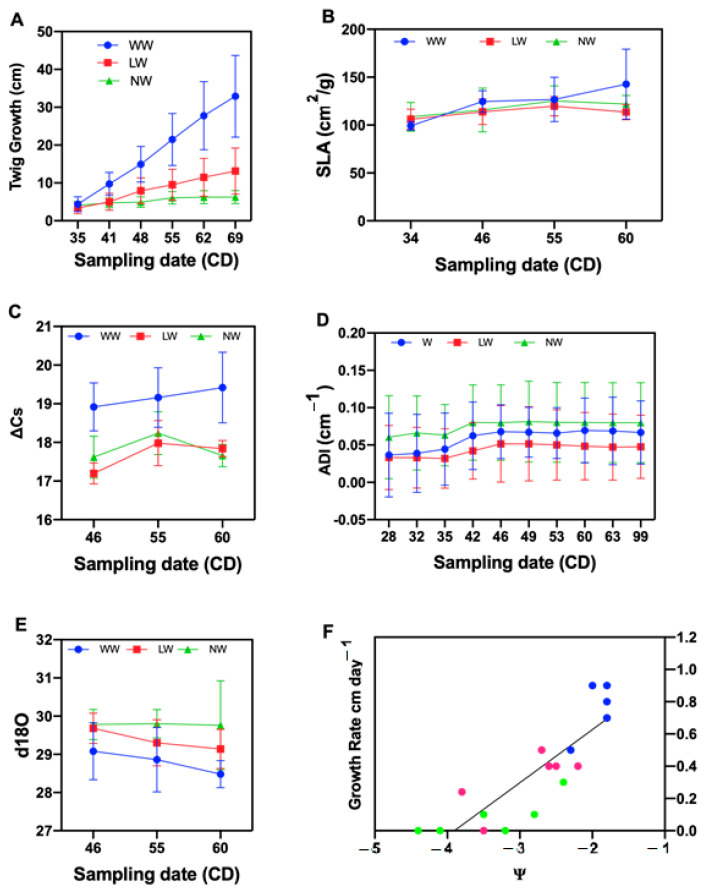
The Y axis shows growth and isotopic discrimination of ^13^C and enrichment of ^18^O as a function of three irrigation conditions between January and March 2016 (X axis). Twig growth (**A**), specific leaf area (**B**), isotopic discrimination of ^13^C (**C**), tamarugo branching architecture (**D**), enrichment of ^18^O (**E**) and leaf water potential vs. twig growth rate under three water level and six sampling dates (**F**). (**F**) is a simple linear regression of twig growth rate vs. mean of predawn and midday water potential corresponding to the same evaluation days. Values represent the mean of twig growth rate for six sampling dates of the evaluation period as a function of mean leaf water potential. Each point represents the mean growth rate of ten plants. The growth rate was calculated through the interpolation and the water potential values represent the mean of predawn and midday leaf water potential together for the same period of evaluation. Blueberry-, strawberry- and lime-colored dots represent well-watered, low watered and non-watered treatments, respectively.

Tamarugo growth rate had a very high sensitivity to water deficit as shown in Figure 1A. Growth detention was observed at between 15 to 20 days in the non-watered plants. By rewatering the non-watered plants with a liter of water, after the growth rate had reached zero in the fourth sampling date, growth resumed (Figure 1A).

The twig growth rate decreased as the leaf water potential decreased, reaching zero at a leaf water potential of −3.16 MPa (Figure 1F), between 8 and 14 February and coinciding with the fourth sampling date, where the growth rate was zero.

There were significant differences between well-watered (WW), low-watered (LW) and non-watered (NW) for specific leaf area. The mean of the well-watered treatment between January and March was higher than the other water levels and statistically significant. There was no significant difference between low-watered and non-watered treatments (Table 1).

A significant difference was observed between no-watered and well-watered treatments. There was no significant difference between well-watered and low-watered treatments for branching architecture, expressed in terms of apical dominance index of tamarugo per water levels. The well-watered and low-watered treatments had an identical behavior for all the sampling dates (Figure 1D). Difference between the first and last sampling date was observed in the well-watered and low-watered treatments (Table 1).

A significant difference was observed for δ^18^O between WW and the other two treatments. There was no significant difference between low and non-watered treatments. A clear enrichment of ^18^O was observed under water stress, indicating stomatal closure. The well-watered presented the lowest value of δ^18^O (Table 1)

A significant difference was observed for Δ ^13^C for the well-watered treatment when compared to the other two treatments (Table 2). There was no significant difference between low- and non-watered treatments. The discrimination of ^13^C was affected by water stress. There was a decline in the discrimination of ^13^C in the low and non-watered together, which can be explained by a partial stomatal closure. The results of the analysis of the discrimination of ^13^C indicate that the photosynthesis is affected by the water stress in tamarugo.

### 2.2. Effect of Water Stress on Tamarugo Water Status and Stomata Functioning

A significant difference was also observed in all the treatments for pre-dawn water potential (Ψpd) and midday water potential (Ψmd) (Table 2). The well-watered treatment showed higher Ψpd and Ψmd values compared to the other water levels (Table 2).

A significant difference between water levels was observed for relative water content (RWC) (Table 2). The well-watered plants showed high RWC values compared to the other water levels.

**Figure 2:**
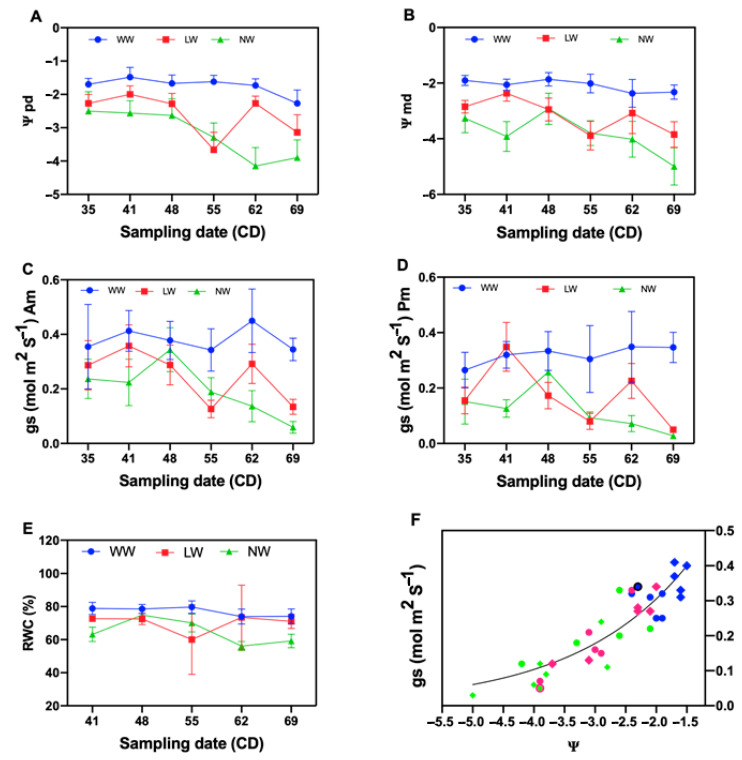
Tamarugo water status and stomata functioning as a function of three irrigation conditions between January and March 2016. leaf predawn water potential (**A**), leaf mid-day water potential (**B**), stomata conductance in the morning (**C**), stomata conductance in the afternoon (**D**), relative water content (**E**) and stomatal conductance vs. leaf water potential under the three water levels during the morning and the afternoon (**F**). Solid diamond shapes represent the mean stomatal conductance on the afternoon vs. midday leaf water potential: the solid blueberry diamond shapes represent the mean of well-watered plants, solid redberry diamond shapes represent the mean of the low-watered plants and solid lime diamond shapes represent the non-watered plants. Solid circle shapes represent the mean of stomatal conductance of the morning vs. predawn leaf water potential: the solid blueberry circle shapes represent the mean of well-watered plants, solid redberry circle shapes represent the mean the low-watered plants and solid lime circle shapes represent the non-watered plants.

There was a high and constant RWC in all sampling dates in the well-watered treatments (Figure 2E). A decrease in RWC was observed as water stress increased. A very low water content (60.02%) in the third sampling date of the medium stress level was observed (Figure 2E).

Significant differences were observed for stomatal conductance (gs) of the morning (a.m.) and stomatal conductance (gs) of the afternoon (p.m.) of *P. tamarugo* under all water levels (Table 2).

## 3. Discussion

### 3.1. Tamarugo Water Status and Stomatal Functioning at Different Water Condition Levels

The results of this study demonstrate that under medium and intense stress, the predawn and midday water potential and the stomatal conductance were lower compared to those in the well-watered treatment. The difference between predawn and midday leaf water potentials was relatively conserved independently of the water condition level. This means that in the experimental conditions, tamarugo was able to maintain the soil-leaf water potential gradient necessary to ensure the flow of water through the continuous soil–plant atmosphere. The average pre-dawn and midday water potential were decreased as the drought progressed.

Tamarugo maintained a relatively high gs for a low Ψl, and only a diminution of 50% of the stomata conductance was observed at −2.6 MPa. The results may confirm the reported tamarugo anisohydric behavior. Other species such as *Acer saccharum*, *Helianthus annus* and *Eucalyptus gomphocephala* have a greater Ψl range of water potential than isohydric species [5,11,18,19,20,21]. A low stomatal conductance was observed for the medium and intense stress conditions at the two measurement periods of the day, which could explain the partial stomatal closing of tamarugo described by Garrido et al. [22] in the condition of reduced water availability, where the minimum water potential measured in leaf at midday was −2.8 MPa, much higher than the value measured in this study. The declining tendency in stomatal conductance of the stressed plants observed suggests a stomatal control of transpiration [5,23] in these plants, to reduce water expenses. In addition to this, Tardieu and Simonneau [5] mention how the water potential of well-hydrated plants fluctuates during the day due to the evaporative demand and stomatal opening, with water potential having its maximum at predawn.

A decline in the discrimination of ^13^C in the low and non-watered treatments was observed, which could be explained by the decrease in the stomatal conductance in the stress conditions. The trees growing without stress had a higher mean value of Δ ^13^C and a lower mean δ^18^O compared to the trees growing in the medium and intense water stress. This higher value of Δ in the well-watered may be linked to a high rate of photosynthesis and stomatal conductance as suggested by Farquhar et al. [24]. Furthermore, the results indicate that under reduced water availability, *P. tamarugo* has lower assimilation (lower Δ ^13^C isotopic discrimination) and a lower integrated gs, indicated by higher values of ^18^O isotopic composition at the leaf level [25]. These results of ^13^C and ^18^O correspond to results found by Garrido et al. [22] in natural tamarugo trees in the Pampa of Tamarugal. The high photosynthetic capacity observed through Δ ^13^C in the well-watered treatment correlates with the high growth rate at the same water level [26,27].

Increased enrichment of ^18^O was observed as the water stress was more intense. The values found in this study (between 28.5 and 29.8‰) are consistent with those reported by Billings et al. [28] in a study of trees of the genus *Quercus* rubra after 30 years of monitoring, where they found that the values of δ^18^O during periods of rainy years ranged between 25.5 and 29.5‰ and for the period of dry years between 26.9 and 31‰. The high value of leaf δ^18^O measured in the medium and intense stress are the result of the low gs values measured on the same date. The low δ^18^O observed in the well-watered treatment are the result of high stomatal conductance recorded between January and March [29].

### 3.2. Tamarugo Growth at Different Water Levels

The results of this study demonstrate that water stress affects the twig growth of *P. tamarugo*, as it affects all species. In the medium water stress, the twig growth rate was lower compared to well-watered. Under intense water stress, tamarugo twig growth decreased along with tamarugo twig water potential. However, Tamarugo has the capacity to grow at low leaf water potential, therefore its growth rate reached zero at a leaf water potential of −3.16 MPa, which could explain the potential of tamarugo to grow under extreme conditions (characteristic of desert plants). According to our results, it is possible to conclude that the tamarugo trees of this study have been affected in twig elongation, which could generate further limitations.

Furthermore, the results of this study demonstrate that water stress affects the specific leaf area of *P. tamarugo* by decreasing the leaf area. The well-watered treatment leaf area values were higher compared to low- and non-watered treatments. According to these results, *P. tamarugo* could regulate its water demand via partial stomatal closure and through leaf area reduction under a water deficit situation. On the other hand, the leaf area was proportional to the leaf dry weight at all water levels. The reduction in the development of leaf area is considered as an early response to water deficit, through which plants tend to reduce their transpiration rates, and thus facilitates the conservation of water [30]. Additionally, the results of this study demonstrate that water stress affects the apical dominance index in the branching architecture of *P. tamarugo*. There were higher values of the apical dominance index in the branching architecture of *P. tamarugo* in the non-watered treatment. There was no significant difference between the first and the last sampling dates among treatments, which can be attributed to the low growth in the height of the branches and no ramification of the tamarugo under water stress, whereas significant differences between the first and the last sampling dates were observed in the well-watered and the low-watered plants. According to the behavior observed for this index (the apical dominance index (ADI)), it is possible to conclude that, being in the optimal conditions, *P. tamarugo* maintains a high number of ramifications per cm of branch. The apical dominance index could be a good monitoring indicator of growth in trees under no optimal water status for conservation purposes.

Hsiao and Acevedo [12] pointed out that at the cell scale, there is a sequence of the process that is affected by the water stress, starting with decreased cell growth, inhibition of cell division, inhibition of wall and protein synthesis, accumulation of solutes, closing of stomata and inhibition of photosynthesis. In addition, Passioura [31] pointed out that at the whole plant scale, there is a sequence of events during a gradual water deficit: (i) decrease in twig growth; (ii) decrease in stomatal conductance, which reduces the rate of CO_2_ assimilation and affects the photosynthesis; (iii) accumulation of solutes in the cells; (iv) decrease in root growth; (v) senescence of leaves, which would lead to a decline of the whole plant. In the current study, the water stress decreased the twig growth of *P. tamarugo*, the specific leaf area and the apical dominance index in the branching architecture of *P. tamarugo*. This reduction observed in these growth variables in the water stress treatments may be the consequence of the reduction in the rate of CO_2_ assimilation. Because the decline in the discrimination of ^13^C, the decrease of the stomatal conductance and the increasing leaf δ^18^O observed in the stress conditions are indexes of the fact that the photosynthetic capacity of the plants was affected by the water stress.

## 4. Materials and Methods

### 4.1. Site Description

The experiment was performed at the Antumapu Experiment Station, Faculty of Agronomy, University of Chile, Santiago, Chile (Metropolitan Region, 33°40′ S and 70°38′ W, 605 m altitude). This is a temperate climate zone with a Mediterranean semiarid climate. The annual temperature varies between 29 °C as the maximum mean in January and 2.8 °C as the minimum mean in July. The long-term mean precipitation is 369.5 mm [32].

### 4.2. Study Design

The experimental unit was a pot with approximately 16.1 L (polypropylenes white tubs of 0.80 m height and 0.16 m diameter) containing homogeneous substrate mix made of 1/3 of sand and 2/3 compost. Plants of tamarugo that were 2–3 years old were planted in these pots in the field. They were acclimated to the site for a month, with the plants being at their optimal condition. Trees with similar morphology were selected for the experiment after the acclimation period. The acclimated plants were subjected to three water stress levels during a period of two months.

Three levels were tested: (i) well-watered (WW), (ii) medium stress intensity (low-watered (LW)) and (iii) intense stress (non-watered (NW)), with 10 replicates for each level. The well-watered condition was maintained between pot capacity and 60% available water, the medium stress was maintained between pot capacity and 20% available soil moisture and plants with the intense stress received only a liter of water during the experimental period after the growth reached zero. The sampling date factor was variable, depending on the frequency of the variable’s measurement.

The establishment of the water treatment was 25 January, when plants under all water treatments were irrigated. The irrigation dates of the WW were 29 January; 1, 5, 9, 12, 16, 19, 23, 26 February and 1, 4 March. The irrigation dates of the LW were 9 and 26 February. The NW received a liter of water after zero growth being recorded on 15 February.

The pot volumetric water content was recorded every two days during the experimental period to determine the watering date using a Decagon soil humidity sensor, Model CS4 (Decagon Devices, Inc. 2365 NE Hopkins Court, Pullman, WA 99163, USA) along with a data logger ProCheck Decagon, Model PC-1 (Decagon Devices, Inc. 2365 NE Hopkins Court, Pullman, WA 99163, USA). The pots had five access points at a 13.5 cm of distance from the top of the pot down covered with scotch tape. The volumetric water content was measured at 10–15, 23–28, 37–42, 50–55 and 64–69 cm depth.

A nutrients application with the commercial formula of the fertilizer was (ANASAC trees and shrubs: N-P-K 11-10-15) at 13 g per plant (expressed as N, PO and KO respectively). This was done a few days after transplanting.

Control of pests, with an approximate frequency of 15 days, was done during the period with 0.1% Dimethoate (ANASAC). The water used for irrigation was drinking water. The water had pH of 7.10 and electrical conductivity of 0.24 mmhos/cm and was low in sodium and carbonates.

### 4.3. Measured Variables

The growth of tamarugo was evaluated in terms of branching architecture, specific leaf area and twig length.

### 4.4. Branching Architecture

The branching architecture was measured at a frequency of one week. A twig that best represented the branching architecture reaching the outer part of the canopy was selected. The base of this branch was the starting point for measuring (1) the total length of the branch, which is the distance from the starting point to the tip of its longest-living terminal, and (2) the number of ramification points that lead to living branches. The indicator of the branching architecture, called apical dominance index (ADI), was obtained by dividing the number of ramifications by the total length of the branch in centimeters [33].

ADI = NR/TLB (cm^−1^) (1)

where ADI is the apical dominance index, NR is the number of ramifications and TLB is the total length of the branch.

### 4.5. Specific Leaf Area

A sample of five leaves per experimental unit was used for the determination of specific leaf area (SLA) at a frequency of one week. Leaf area was determined using photography and software (Image J) as described by [34]. Immediately after taking the picture for leaf area determination, the sample was put into an oven at 60 °C for 48 h to determine the dry weight.

The specific leaf area was calculated using the following:

SLA = LA/LDW (cm^2^ g^−1^) (2)

where SLA is the specific leaf area, LA is the leaf area and LDW is the leaf dry weight.

### 4.6. Twig Growth Rate (TGR)

The length of two twigs of each pot was measured at a frequency of 3 and 4 days using a ruler. The twigs were measured from the starting point to the tip. The first measurement was considered as reference or time zero. The first twig (TL_1_) was calculated by subtracting the twig length of the first length measurement from the twig length of the second measurement to guarantee a uniform twig length data.

The twig growth rate was calculated with the data obtained during the experiment period, as:

(TL_j_ − TL_j−1_)/(T_j_ − T_j−1_) (cm/day), (3)

where TL_j_ is twig length at Tj, TL_j−1_ is the twig length at Tj_−1_, T_j_ is the last measurement time and T_j−1_ is the first measurement time.

### 4.7. Relative Water Content (RWC)

The RWC was measured as described by [35]: the leaf material was weighed immediately after sampling to take a fresh weight. Later, the measurement of the fully turgid weight of the tissue was taken by weighting the samples after placing them in petri dishes containing distilled water in the laboratory at ambient light and temperature for 24 h and removing any excess water on their surface using an absorbent tissue. Later, the samples were dried at 70 °C for 24 h before reweighing for the dry weight. The RWC was calculated at a frequency of one week according to:

RWC = (FW − DW)/(TW − DW) × 100(4)

where FW is the fresh weight, DW is the dry weight and TW is the fully turgid weight.

### 4.8. Predawn Leaf (Ψpd) and Midday Leaf Water Potential (Ψmd)

The leaf water potential was measured with a Wescor’s psychometric sample chamber (Wescor model C-52) connected to a automated eight-channel Microprocessor controlled water potential datalogger (Wescor model PSYPRO, WESCOR Inc USA) in leaflets taken at pre-dawn and at mid-day. The predawn measurements were done at the end of the night, before dawn, between 3 and 6 a.m. And the mid-day leaf water potential was measured in samples taken close to solar noon, from 2:40 to 3:30 p.m. local time.

### 4.9. Stomata Conductance

Stomata conductance was recorded for six weeks with a weekly frequency, between 8 and 10 a.m.; and between 2 and 4 p.m. using a leaf porometer Decagon, Model SC-1 (Decagon Devices, Inc. 2365 NE Hopkins Ct. Pullman, WA 99163 USA). For the measurements, the leaflets of the composite leaf were positioned in the porometer chamber, taking four measurements on four leaves of different branches of each plant.

### 4.10. Isotopic Discrimination of ^13^C and Enrichment of ^18^O

Leaf samples were taken three times during the period of evaluation between January and March 2016. At each sampling date, a leaf sample of 20 g approximately was taken from two pots (plants) of the same water level (n = 5 samples per water level). Each sample was dried at 60 °C for 48 h in a forced-air oven (Venticell, MMM Group, Planegg/Munchen, Germany) until constant weight. The samples were then crinkled to a homogeneous powder with a power mill. Two sub-samples were taken from each sample, weighted with an analytical balance (Precisa 125 A, Precisa Instruments, Dietikon, Switzerland) and put in tin (0.002 ± 0.003 g) and silver (0.0008 ± 0.001 g) capsules to measure isotopic composition of ^13^C (δ^13^C) and ^18^O (δ^18^O), respectively. Analyses were performed by the Stable Isotope Laboratory at the Agronomy Sciences Faculty of the University of Chile with an Isotope-ratio mass spectrometer (IRMS) model INTEGRA2 (SERCON Ltd. Cheshire, UK) based on the high performance 20–20 stable isotope analyzer and ANCA-SL sample preparation module with a precision of 0.3‰ and 0.5‰ for ^13^C and ^18^O, respectively. As a reference, wheat flour OAS (SC0464, SERCON Ltd. Cheshire, UK) of known isotopic composition (δ^13^C = −28.01 ± 0.12‰ and δ^18^O = 32.27 ± 1.2‰) and (δ^13^C = −25.64 ± 0.17‰ and δ^18^O = 28.51 ± 0.2‰) was used as the internal control of *P. tamarugo.*

### 4.11. Statistical Analysis

Data were graphed using *GraphPad Prism* version 8.0.0 for Mac OS X, GraphPad Software, La Jolla California USA, www.graphpad.com and were analyzed using InfoStat. The data were analyzed through analysis of variance using a mixed model with measurements repeated in time by considering water condition and sampling date. The analysis of variance (ANOVA) was performed by running a General Linear Model (GLM) method and the traditional ANOVA process. A α ≤ 0.05 was chosen for all the ANOVAs performed in this study.

## 5. Conclusions

Tamarugo has anisohydric behavior when facing water stress: tamarugo lets the leaf water potential decrease as the soil water potential decreases, with a partial stomatal closure strategy reflected in the tendency to decrease the stomatal conductance and increase ^18^O Tamarugo can maintain a high stomatal conductance at low leaf water potential. In addition, tamarugo reduces its leaf area as a strategy to diminish the water demand. Furthermore, as growth is particularly sensitive to changes in cell turgor associated with water condition, tamarugo twig growth decreases along with the leaf water potential of the tree.

## Figures and Tables

**Table 1:** Summary analysis of tamarugo growth parameters, Isotopic Discrimination of ^13^C and Enrichment of ^18^O under three water conditions. Means and standard errors (±S.E.) of twig daily growth rate, specific leaf area (SLA), branching architecture (BA) expressed as Apical Dominance Index (ADI), isotopic discrimination of ^13^C (Δ ^13^C) and enrichment of ^18^O (δ^18^O) of *P. tamarugo* observed under three water levels.

Growth Parameters/Water Conditions	TGR (cm day^−1^)		SLA (cm^2^ g^−1^)		BA (ADI) (cm^−1^)		Δ ^13^C (‰)		δ^18^O (‰)	
WW	0.80 ±0.06	a	123.3 ±3.14	a	0.06 ±0.01	b	19.2 ±0.15	a	28.8 ±0.17	b
LW	0.34 ±0.06	b	113.3 ±3.14	b	0.04 ±0.01	b	17.7 ±0.15	b	29.4 ±0.17	a
NW	0.18 ±0.06	c	117.9 ±3.14	b	0.08 ±0.01	a	17.8± 0.26	b	29.8± 0.17	a

Different letters indicate significant difference for water conditions according to the DGC test (α = 0.05). WW: mean of well-watered; LW is the mean of low watered and NW is the mean of no watered.

**Table 2:** Summary analysis of tamarugo water status and stomata functioning parameters under three water conditions. Means and standard errors (±S.E.) of predawn leaf water potential (PD Ψ) (MPa), midday leaf water potential (MD Ψ) (MPa), leaf relative water content (RWC) (%), a.m. stomatal conductance (am gs) (mol m^2^ S^−1^) and p.m. stomatal conductance (pm gs) (mol m^2^ S^−1^) of *P. tamarugo* under three water levels.

Water status Parameters/Water Conditions	PD Ψ (Mpa)		MD Ψ (Mpa)		RWC (%)		am gs (mol m^2^ S^−1^)		pm gs (mol m^2^ S^−1^)	
WW	−1.75 ± 0.07	a	−2.09 ± 0.09	a	77.04 ± 1.65	a	0.38 ± 0.01	a	0.32 ± 0.01	a
LW	−2.60 ± 0.07	b	−3.17 ± 0.09	b	69.94 ± 1.65	b	0.25 ± 0.01	b	0.17 ± 0.01	b
NW	−3.17 ± 0.07	c	−3.82 ± 0.09	c	64.70 ± 1.65	c	0.20 ± 0.01	c	0.12 ± 0.01	c

Different letters indicate significant difference for water condition, according to the DGC test (α = 0.05). WW is the mean of well-watered, LW is the mean of low watered and NW is the mean of no watered. a.m. is the abbreviation of **ante meridiem** and p.m. is the abbreviation of **post meridiem**, that is, noon and afternoon, respectively.

## Data Availability

The data presented in this study are available on request from the corresponding author. The data are not publicly available due to restrictions eg privacy or ethical.

## References

[1] Calderon G., Garrido M., Acevedo E. (2015). Prosopis tamarugo Phil.: A native tree from the Atacama Desert groundwater table depth thresholds for conservation. Rev. Chil. Hist. Nat..

[2] Williams A.P., Allen C.D., Macalady A.K., Griffin D., Woodhouse C.A., Meko D.M., Swetnam T.W., Rauscher S.A., Seager R., Grissino-Mayer H.D. (2013). Temperature as a potent driver of regional forest drought stress and tree mortality. Nat. Clim. Chang..

[3] Frelich L. (2002). Forest Dynamics and Disturbance Regimes: Studies from Temperate Evergreen-Deciduous Forest.

[4] Rojas R., Dessargues A. (2007). Groundwater flow modelling of the regional acquifer of the Pampa del Tamarugal, northern Chile. Hydrogeol. J..

[5] Tardieu F., Simonneau T. (1998). Variability among species of stomatal control under fluctuating soil water status and evaporative demand: Modelling isohydric and anisohydric behaviours. J. Exp. Bot..

[6] Rood S.B., Zanewich K., Stefura C., Mahoney J.M. (2000). Influence of water table decline on growth allocation and endogenous gibberellins in black cottonwood. Tree Physiol..

[7] Horton J.L., Kolb T.E., Hart S.C. (2001). Physiological response to groundwater depth varies among species and with river flow regulation. Ecol. Appl..

[8] Cooper D.J., D’Amico D.R., Scott M.L. (2003). Physiological and morphological response patterns of Populus deltoides to alluvial groundwater pumping. Environ. Manag..

[9] Lambers H., Champin F.S.I., Pons T.L., Lambers H., Chapin F.S., Pons T.L. (1998). Photosynthesis, respiration, and long distance transport. Plant Physiological Ecology.

[10] Baohua G., Ying G., Meiying F., Xiaoyin N., Yijun L., Jie C. (2003). Phenotypic plasticity of growth and morphology in Mosla chinensis responds to diverse relative soil water content. Acta Ecol. Sin..

[11] McDowell N., Pockman W.T., Allen C.D., Breshears D.D., Cobb N., Kolb T., Plaut J., Sperry J., West A., Williams D.G. (2008). Mechanisms of plant survival and mortality during drought: Why do some plants survive while others succumb to drought?. New Phytol..

[12] Hsiao T.C., Acevedo E. (1974). Plant responses to water deficits, water-use efficiency, and drought resistance. Agric. Meteorol..

[13] Flexas J., Diaz-Espejo A., Gago J., Gallé A., Galmés J., Gulías J., Medrano H. (2014). Photosynthetic limitations in Mediterranean plants: A review. Environ. Exp. Bot..

[14] Time A., Garrido M., Acevedo E. (2018). Water relations and growth response to drought stress of Prosopis tamarugo Phil. A review. J. Soil Sci. Plant Nutr..

[15] Lehner G., Delatorre J., Lütz C., Cardemil L. (2001). Field studies on the photosynthesis of two desert Chilean plants: Prosopis chilensis and Prosopis tamarugo. J. Photochem. Photobiol. B Biol..

[16] Delatorre J., Pinto M., Cardemil L. (2008). Effects of water stress and high temperature on photosynthetic rates of two species of Prosopis. J. Photochem. Photobiol. B Biol..

[17] Chávez R.O., Clevers J.G.P.W., Herold M., Acevedo E., Ortiz M. (2013). Assessing water stress of desert tamarugo trees using in situ data and very high spatial resolution remote sensing. Remote Sens..

[18] Barnes F.J. (1987). Carbon Gain and Water Relations in Pinyon-Juniper Habitat Types (Photosynthesis, Water Stress, Gradient Analysis, Ecophysiology).

[19] Loewenstein N.J., Pallardy S.G. (1998). Drought tolerance, xylem sap abscisic acid and stomatal conductance during soil drying: A comparison of canopy trees of three temperate deciduous angiosperms. Tree Physiol..

[20] Franks P.J., Drake P.L., Froend R.H. (2007). Anisohydric but isohydrodynamic: Seasonally constant plant water potential gradient explained by a stomatal control mechanism incorporating variable plant hydraulic conductance. Plant Cell Environ..

[21] West A.G., Hultine K.R., Sperry J.S., Bush S.E., Ehleringer J.R. (2008). Transpiration and hydraulic strategies in a piñon-juniper woodland. Ecol. Appl..

[22] Garrido M., Silva P., Acevedo E. (2016). Water relations and foliar isotopic composition of prosopis tamarugo phil., an endemic tree of the atacama desert growing at three levels of water table depth. Front. Plant Sci..

[23] Valladares F., Vilagrosa A., Peñuelas J., Ogaya R., Camarero J., Corcuera L., Sisó S., Gil-Pelegrín E. (2004). CAPÍTULO 6 Estrés hídrico: Ecofisiología y escalas de la sequía. Water.

[24] Farquhar G.D., O’Leary M.H., Berry J.A. (1982). On the relationship between carbon isotope discrimination and the intercellular carbon dioxide concentration in leaves. Aust. J. Plant Physiol..

[25] Hasselquist N.J., Allen M.F., Santiago L.S. (2010). Water relations of evergreen and drought-deciduous trees along a seasonally dry tropical forest chronosequence. Oecologia.

[26] Poorter H., Remkes C. (1990). Leaf area ratio and net assimilation rate of 24 wild species differing in relative growth rate. Oecologia.

[27] Lambers H., Poorter H. (1992). Inherent Variation in Growth Rate Between Higher Plants: A Search for Physiological Causes and Ecological Consequences. Adv. Ecol. Res..

[28] Billings S.A., Boone A.S., Stephen F.M. (2016). Tree-ring δ13C and δ18O, leaf δ13C and wood and leaf N status demonstrate tree growth strategies and predict susceptibility to disturbance. Tree Physiol..

[29] Barbour M.M. (2007). Stable oxygen isotope composition of plant tissue: A review. Funct. Plant Biol..

[30] Westgate M.E., Boyer J.S. (1985). Osmotic adjustment and the inhibition of leaf, root, stem and silk growth at low water potentials in maize. Planta.

[31] Passioura J.B. (1996). Drought and drought tolerance. Plant Growth Regul..

[32] Novoa R., Villaseca S., del Canto P., Sierra C., del Pozo A. (1989). Mapa Agroclimático de Chile.

[33] Pérez-Harguindeguy N., Diaz S., Gamier E., Lavorel S., Poorter H., Jaureguiberry P., Bret-Harte M.S., Comwell W.K., Craine J.M., Gurvich D.E. (2013). New handbook for standardised measurement of plant functional traits worldwide. Aust. J. Bot..

[34] Mitchell P.J., O’Grady A.P., Tissue D.T., White D.A. (2013). Drought response strategies define the relative contributions of hydraulic dysfunction and carbohydrate depletion during tree mortality. New Phytol..

[35] Pietragalla J., Mullan D. (2012). Leaf relative water content. Physiological Breeding II: A Field Guide to Wheat Phenotyping.

